# A Review of the Real-Time Monitoring of Fluid-Properties in Tubular Architectures for Industrial Applications

**DOI:** 10.3390/s20143907

**Published:** 2020-07-14

**Authors:** Maha A. Nour, Muhammad M. Hussain

**Affiliations:** 1 mmh Labs, Electrical Engineering, Computer Electrical Mathematical Science and Engineering Division, King Abdullah University of Science and Technology (KAUST), Thuwal 23955-6900, Saudi Arabia; maha.nour@kaust.edu.sa; 2 EECS, University of California, Berkeley, CA 94720, USA

**Keywords:** fluid sensors, pipes, tubes, flowmeter, viscosity monitor

## Abstract

The real-time monitoring of fluid properties in tubular systems, such as viscosity and flow rate, is essential for industries utilizing liquid mediums. Nowadays, most studies of the fluid characteristics are performed off-line using laboratory facilities that can provide accurate results, yet they do not match the demanded industrial pace. Off-line measurements are ineffective and time-consuming. The available real-time monitoring sensors for fluid properties are generally destructive methods that produce significant and persistent damage to the tubular systems during the installation process. Others use huge and bulky invasive instrument methods that generate considerable pressure reduction and energy loss in tubular systems. For these drawbacks, industries centered their attention on non-invasive and non-destructive testing (NDT) methodologies, which are installed on the outer tubular surface to avoid flow disturbance and desist shutting down systems for installations. Although these sensors showed excellent achievement for monitoring and inspecting pipe health conditions, the performance was not convincing for monitoring the properties of fluids. This review paper presents an overview of the real-time monitoring of fluid properties in tubular systems for industrial applications, particularly for pipe monitoring sensors, viscosity, and flow measurements. Additionally, the different available sensing mechanisms and their advantages, drawbacks, and potentials are discussed.

## 1. Introduction

Fluid manufacturing industries, such as paints, petrochemicals, oil and gas, beverages, and medicines, need safe and efficient operation with optimized resource management. One of the critical factors for fluid industries is the monitoring of fluid properties at several manufacturing stages using real-time monitoring sensors for tubular systems [1]. Off-line and delayed measurements are undesirable for the fast-paced industrial economy for several reasons. First, delayed measurements cause high maintenance costs for improper performance and disturbance to fluid supply [2,3,4]. It is time-consuming to send samples to a laboratory for an examination that might take several days due to fluids sample preparations, as well as possible required safety and health precaution procedures [5]. Additionally, delayed measurements might provide poor results compared to real-time analysis of fresh samples. The fluid characteristics of stored samples might alter, influenced by the surrounding environment of the storage conditions, such as exposure to elevated temperature, humidity, or light until the analysis time [6]. As a result, continuous and real-time monitoring of fluid properties is fundamental for fluid industries to allow precise production control, informed decision making, and cut maintenance expenses. 

It is essential to understand the fluidic environment and its impact on the associated industrial resources [7]. However, most of the available fluid sensors are off-line and used for laboratory setup analysis, which is inefficient and time-consuming. Furthermore, the used technologies for fluidic monitoring applications in the industry are not synchronized with up-to-date technologies nor meeting the ever-increasing demands. While having an integrated sensory system that can monitor the intended stimuli is needed, it is equally challenging to achieve multi-functionality in a single platform system, especially since all the known electronic components functioning in the fluid environment are rigid, bulky [8], and not compatible with tubular curvature architecture. Thus, a compliant version that is physically flexible is needed more than ever to be deployed conveniently in locations with different tubular diameters and dimensions, without disturbing the fluid flow, or generating pressure drop or energy loss. However, the main challenge is that most of the electronics, and particularly integrated electronic systems, are made with rigid and brittle materials (mainly silicon). Therefore, it is vital to develop integration strategies to achieve a fully compliant integrated electronic system. Moreover, it is urged for price affordability to use low-cost materials and fabrication processes.

The objective of this review is to provide a summary of up-to-date available technologies for real-time monitoring sensors of fluid properties in tubular systems, such as pipes, especially of mechanically flexible platform sensors. Focuses on real-time monitoring sensors for fluid properties or pipe health conditions, which can provide continuous or periodically instant results versus time. In the worst-case scenarios, it considers up to a few minutes to allow a fast response, where it does not require samplings or in situ testing. The literature review covers the available technologies for pipe monitoring sensors, flowmeters, and viscometers. The pros and cons and main challenges of the available technologies are discussed. The study is directed towards incompressible Newtonian fluids.

## 2. Pipe Monitoring Sensors

Real-time monitoring and periodic assessments for pipe systems are an essential routine for industries and countries’ infrastructures, to protect their properties and prevent the hazardous risk that might cause as life-threatening to humans and the surrounded environment. Leaking of radioactive waste from nuclear plants or a pipe explosion from highly pressured pipes are examples of the danger for inappropriate diagnostics of pipelines [9]. Exposing pipes to harsh environments, such as elevated temperature for power plants or high pressure in the oil and gas industry or corrosive chemicals, increase the aging and failures of the system leading to more frequent and strict evaluation and integrity management practice for safe operation [10,11].

The most common types of pipe inspection technology are non-invasive testing and non-destructive testing (NDT) methods. The NDT monitors pipelines without generating permanent damage to pipes, such as the pigging technology. The non-invasive methods monitor the pipe conditions without disturbing the fluid’s flow inside the pipes, such as attaching the acoustic sensors probes to the tubes’ outer surface. For that reason, NDTs and non-invasive methods are the most convenient inspection technologies. Their excellent and reliable performance has been proven for several years by fluidic industries. 

**Figure 1:**
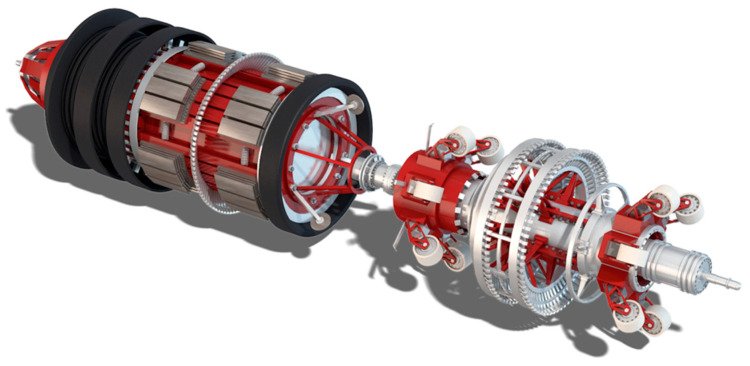
Intelligent pig gauge for internal pipe inspection from the open-source image library of Nord Stream AG.

Nowadays, there are real-time and non-destructive testing (NDT) monitoring sensors for pipe applications, specifically for pipe health inspection of conditions from corrosions [12] and cracks [13]. Pigging instruments is a primary internal pipe cleaning and inspection technology. It is a plug gauge referred to as a pig that drives through the pipes and performers several maintenance activities, including mechanical cleaning with brushes, detecting corrosions, measures internal pipe diameters, and identifies defects locations [14]. It started in the late 1800s as a pipe cleaning process [15]. Since then, it has been developed to a smart pig tool as it has integrated with sensors, cameras, and brushes, for improving inspection technologies and enhancing the cleaning process [16]. Figure 1 shows an intelligent pig gauge for internal pipe inspection from the open-source image library of Nord Stream AG. The technique requires launcher stations for loading the pigs into the pipes and receiver stations to unload them from the pipes. It travels with the product flow through the pipes for real-time measurements without suspending the pipes’ fluid flow [15]. Although pigging technology is an essential process for pipes maintenance, it is still not an easy choice. Introducing such a bulky and large tool into the industrial pipeline is a risky procedure [17]. Technical problems and system failure have not been eliminated, including a stuck or lodged pig gauge from clogged pipes with wax [18]. Such a problem has a significant negative impact, including blocking product flows, rising expenses from a complete shutdown system, and instrument retrieving [19]. 

Monitoring tubular health conditions using non-invasive and NDT testing limits unnecessary shutdowns, such as those that come from device installation and frequent maintenance when destructive test methods are used. Using these methods prevents unrequired pressure drop formation or fluid flow disturbance inside the tubular systems as installation of bulky measurement instruments within the tube is not required. A network of acoustic sensors is an example of a non-invasive and NDT technology used to monitor tube reliability for industrial applications. Using distributed acoustic sensors along the pipe at discrete distances acoustic emissions can be used to detect cracks and leaks [20,21]. Similarly, other types of sensors can be used, such as ultrasonic sensors [22,23], radiography monitoring [24,25], and eddy current [26].

The authors of [27] reviewed the different pipe monitoring and leaking detection technologies. They classified the developed techniques into three categories: the exterior methods, the visual or biological approaches, and the internal methods. The interior method detects system leakage depending on computational analysis of the internal fluid monitoring instruments such as pressure, flow, density, temperature. For example, leak detection can be noticed from a sudden change in the pressure value or a change in mass or fluid volume. The visual and biological detection methods use cameras on pigging systems, drones, helicopters, or even trained dogs or personnel. Exterior detections are the NDT and non-invasive ways that can achieve their task externally of the pipe systems. Acoustic sensors, accelerometers, fiber optics [28,29], infrared thermography [29,30], fluorescent and electromechanical impedance are some examples of leak detection exterior technologies. In the case of pipe leaking, the acoustic sensor generates elastic waves up to 1 MHz [30], due to the significant pressure drop from fluid releases to the ambient. The infrared thermography is a thermal camera supported by image processing to analyze the leaking remotely through a sudden temperature change. The fluorescent leaking spills detection is limited to fluorescence fluids, such as hydrocarbons. They detected the discharged fluorescence signal after exposing it to a specific light wavelength compatible with the detected fluid. 

Another pipe monitoring technique is the Transient Test-Based Technique (TTBT) for leak detection and fault alarms. It is based on analyzing the measured pressure sensor signal in either the time or frequency domain, which is referred to as the pressure signal [31,32]. It is suitable for the transmission mains (TM), where a sudden change in the pressure signal might be a direct indicator of the system failure, unlike the case for the distribution network [32]. TTBT is an inexpensive measurement technique that requires only a few minutes for the test [9]. Another advantage is that the leaking size, location, and the initial pressure can be determined by analyzing the pressure signal only [33]. A network of distributed pressure sensors and thermometers on the external pipe walls can detect pipe failures. The study [34] presents a relative temperature difference between the pipe wall and the soil at each node from the distributed sensors, where it indicates the unexpected change in the fluid flow rate leading to leaking detection. In addition, each node includes a pressure gauge to detect abnormal pressure drop to support the detection results.

Studying the vibrational response of the pipe characteristics is an approach for damage detection. The change in the vibrational response of pipe acceleration can be an indicator of a change in the physical pipe condition [34]. Monitoring the surface acceleration of pipe under ambient enclosed flow detects the leakages and estimate the severity of the failure status. The paper [35] addresses cross-correlation of surface acceleration for leak detection along the pipe at discrete locations. It provides continuous monitoring by studying the cross-spectral density of the external pipe surface.

Currently, with the growth of flexible electronics, non-invasive and NDT sensors have also been improved and implemented as mechanically flexible probes, such as the flexible eddy current [26,36], ultrasonic [37,38], and acoustic emission probes [39]. Even though these sensors do not interfere with the fluid inside the tubes the development towards mechanically flexible probes provide beneficial features, such as adaptability to the curved surfaces of pipes and elimination of bulky, large sensors. For example, eddy current probes are used on the outer surface of tubes to monitor the inner surface wall for defects and cracks in the pipe systems. The flexible eddy current sensors showed enhanced properties, compared to conventional probes, since it is inexpensive, disposable, and uncomplicated to produce. Moreover, energy harvesting based on piezoelectric materials monitoring sensors capture attention, due to their self-powered or significantly low-power consumption advantages. piezoelectric phenomena allow the sensing of pipe stresses from the external surface, as non-invasive and NDT, by generates charges in the material represented as an increment in the voltage signals under physical deformation or stress. The authors of [40] presented a flexible sensor for monitoring leakages in a water pipe system using PVDF piezoelectric material that allows flexibility for matching the curved pipe nature. Another example of NDT and non-invasive testing is the magnetostrictive monitoring transducer. It depends on the magnetostrictive principle, which describes the mechanical change in ferromagnetic materials as a change in the generated magnetic field on the pipe [40]. One of the main advantages of magnetostrictive sensors applicable for wiring free transducers, making it suitable for elevated pipes temperatures applications [41]. The authors of [42] demonstrated a promising flexible magnetostrictive transducer patch for a real-time monitoring pipe system.

Some pipe monitoring sensors developed to withstand harsh fluids environments, such as corrosive mediums, elevated temperature, and pressure. High-temperature ultrasonic probes improved to withstand elevated pipe temperatures up to 490 °C [43,44]. In addition, the Fibre Bragg Grating (FBG) sensors are used as pressure or temperature sensor for corrosive mediums, as it is insensitive to corrosion [45]. Remote detection methods used for hot fluids and harsh environments such as infrared thermography [29,30]. The harsh environment effected on pipes’ health is not limited to the fluid status but also from the surrounded state. For example, subsea pipelines exposed to high pressure from the water depths, corrosive seawater, and sometimes a significant temperature difference between transferred fluid and surrounded water temperature generating stress on pipes walls increase the risk of failures and monitoring challenges. The review paper [46] discusses the challenges for subsea pipelines and addressed technologies for monitoring applications, including magnetic flux leakages (MFL), eddy current, vibrational sensors, guided wave testing (GWT), fiber optics, and radiography. However, most of the efforts have been made for monitoring pipe conditions, and not the fluid characteristics within the pipes. Therefore, there is still an essential need to develop mechanically flexible real-time sensors to replace the bulky and huge monitoring sensors of fluid properties.

On the other hand, it is evident that invasive and destructive testing methods are not recommended for tubular monitoring applications, because of their significant drawbacks. Specifically, generating permanent damage to industrial tubes by methods such as drilling out piece of the pipe, inserting bulky inspection tools or branching the tubular pathway [11,47,48,49]. Another main disadvantage is disturbing the transferred fluid inside the tubular system by causing permeant pressure drop, energy loss, and budget rise. That might explain the reason for lots of focus on non-invasive and NDT methodologies for pipe monitoring applications, and the limited available technologies for real-time monitoring of fluidics in tubular systems. After all, fluidic monitoring sensors have greater need to be in contact with the fluid for accurate results, as compared to pipe inspection sensors. 

## 3. Pipes Flow Sensors

The real-time monitoring of the real-time flow rate in tubular systems, such as pipes, is vital for fluidic industries, including agriculture, petroleum [50], medicines and chemicals [51], fluids transportation, and water desalination [52,53,54]. It assists in determining the performance of applications for industries, such as an indicator for a product quality control [55], process analysis [56], efficient power control, material utilization including the waste management [57], and estimating the consumption of fluidic products. Table 1 shows a comparison of different flowmeters technologies for pipe systems.

**Figure 2:**
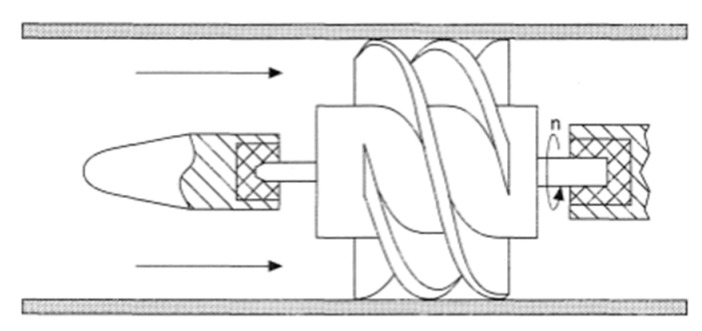
Turbine flowmeter and n is the rotative speed of the turbine [72].

Numerous varieties of real-time flowmeters have been developed amidst the expansion of fluidic industries. Some of the developed technologies include differential pressure [58], thermal [59,60], turbine [61,62], electromagnetic [63,64], vortex [65,66], ultrasonic sensors [67,68], and Coriolis [69,70]. Volumetric and mass are the two types of flowmeters. The turbine flow sensor is a volumetric mechanical flow rate measurement [71]. Figure 2 shows a cross-section area for a turbine flowmeter installed inside a pipe. It works by rotating the turbine’s blades with a rotor speed directly proportional to the flow rate of the pipe fluid [72]. The curved and smooth design of rotor blades has a positive effect on reducing fluid disturbance. However, maintenance problems might arise, due to mechanical failures, such as damaged blades or stopped rotors [73].

**Figure 3:**
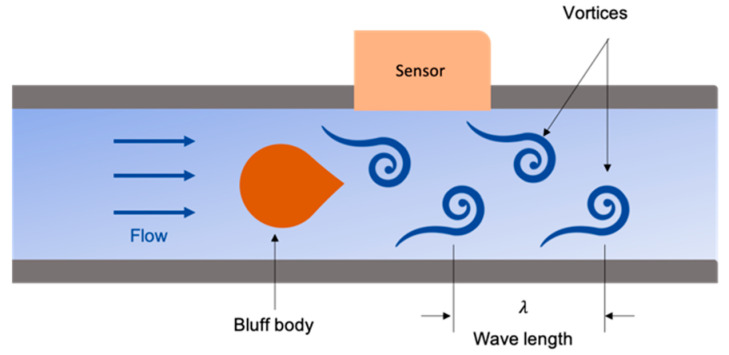
Vortex flowmeter operating principle using a bluff body to generate a repetitive periodic of vortices measured using an external sensor.

Different type of flow sensors is a mass flowmeter. An example of this flow sensor type is a Coriolis flowmeter. It involves a vibrational tube due to the flow force of the fluid acting on the tube’s wall, referred to as the Coriolis force [74]. The Coriolis force forms an anti-symmetric tube shape [75]. Then, the mass flow rate can be detected as a change in the resonance frequency from the vibrational tube [76]. Coriolis provides accurate measurements, since it is independent of pressure, temperature, viscosity, and density. The main drawback of this technology is the pressure drop generated from the fluid flow into the sensing tube section from the original pipe. Another example of a mass flowmeter is the Vortex sensor. It is, likewise, independent of the fluid physical properties [77]. The vortex flowmeter is a simple instrument with economical cost and low maintenance. It involves a bluff body, like a cylinder, to disturb the fluid flow and forming a Karman vortex street phenomenon, which is a periodic creation of swirling vortices [65,71], as shown in Figure 3. The wavelength is consistent among generated vertices at a steady flow rate. The number of vortices formation and their strength are proportional to the fluid flow rate that can be detected with varieties of sensors, including micro-electromechanical system (MEMS) [78], pressure change [79], and piezoelectric sensors [80]. Yet, these flowmeters remain firm, large, and incompatible with the curved nature of the tubes architecture, where their bulkiness disturbs and produces turbulence within the initial flow rate. As a result, they form undesired permanent and significant pressure drop in the tubular systems, which results in increased power consumption to re-establish the required pressure for fluid transfer. Therefore, non-invasive flowmeters are capturing the industry’s attention, since they can overcome the main challenge of bulky flowmeters by being fitted onto the outside wall of a pipe. Furthermore, NDTs and non-invasive are especially of interest after their successful progress in the application of tubular integrity inspections.

**Figure 4:**
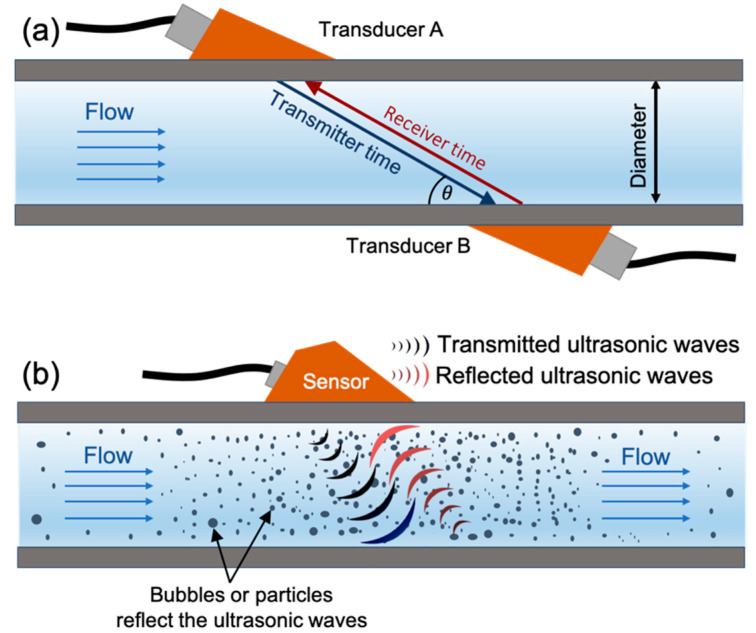
Different ultrasonic flowmeters types. (**a**) Transit time ultrasonic flowmeter. The fluid flow velocity depends on the tube diameter, the transmitter time for ultrasonic wave travels from transducer A to B, the receiver time, and the angle between the transducer and pipe θ. (**b**) Doppler ultrasonic flowmeter serves fluids with suspended bubbles or particles to reflect the transmitted ultrasonic wave.

Electromagnetic and ultrasonic flowmeters are common examples of NDT and non-invasive flowmeters. The electromagnetic flow sensor is based on measuring a change in voltage that is directly proportional to the fluid velocity under an applied magnetic field [64]. However, such technology is restricted to electrically conductive fluids and pipes with an electrical insulating surface. Ultrasonic flow sensors use acoustic vibrations to measure flow rate. They come in two different types, transit time or Doppler shift [81]. The transit time-based ultrasonic flowmeter uses the variation in transit time of an ultrasonic signal between the transmitter and receiver transducers located and aligned on opposite sides of the pipe to determine the fluid flow rate, as shown in Figure 4a. The transient time between the transducers is shorter in the direction of the flow and longer on the direction opposite to the flow. The differential transient time between the upward and downward, Δt, is directly proportional to the flow velocity, v, in the pipe, as shown in Equation (1) [50]. Where L is separated distance between transducers, θ is the angle between the transducer and pipe wall, and c is the wave speed in the flow medium. The configuration of the transducers will depend on several factors such as pipe diameter, space availability, or fluid characteristics. In fact, this method does not work for fluids with lots of bubbles or solid particles nor does it work for partially filled tubes. The accurate positioning of the transducers is critical for accuracy of measurements from a transit time ultrasonic flowmeter [55].

(1) $$\Delta t=\frac{2\;v\;L\;cos\theta }{c^{2}-v\;cos^{2}\theta }$$

In contrast, Doppler ultrasonic flow meters are easy to install, since no alignments are required, as the transmitter and receiver transducers are located on the same device, as shown in Figure 4b. It operates by emitting an ultrasonic wave through a tubular system, the wave reflects off bubbles or particles in the fluid medium and shifts the frequency of the wave, which is measured at the receiving transducer [82]. Doppler effect flow meters are limited for fluids containing solid particles or bubbles with a flow rate high enough to keep them suspended, and only work on partially filled tubes when the transducers are installed below the liquid level. 

Although several different types of pipe flowmeters are available, they are not yet fully optimized and convenient, due to the limitations discussed. There is still a demand for the development and improvement of NDT and non-invasive flow sensor technologies for monitoring tubular systems in industrial applications. During the last decade, there has been significant progress and improvements of flow meters used for real-time monitoring of microchannels that might be applied for use in macro dimensional tubular systems of industrial applications.

### Microfluidic Flow Sensors

Microfluidic flow sensors have been developed robustly in the last years for measuring the flow rate in small volumes, such as in biomedical applications [82,83] and analytical chemistry applications [84]. MEMS [85], optical [86,87] thermal [88,89,90], or pressure-based measurement flow sensing technology [91,92], are some examples of robust developed microfluidic flow sensors. 

The common structures for MEMS flowmeters in microchannels are a cantilever [93], membrane [94], and the Coriolis flowmeters, which is a suspension and vibrational microchannel [95]. The membrane or the cantilever deflection in microchannels is interpreted directly into the channel flow rate, where the applied pressure from the flowrate is directly proportional to the cantilever surface area [96]. The cantilever deflection is detected using various methods, such as using the support of optical techniques [97,98,99] or electrical methods, including the capacitive cantilever [100,101], piezoelectric [102,103], the piezoresistive effect [104,105], and magnetic materials [106]. The MEMS Coriolis structures determine the mass flow rate based on the change in Coriolis forces in a vibrating channel or tube [107]. The mass flow rate Q is inversely proportional to the resonating frequency of the tube ω, as expressed in Equation (2) [95]. K_s_ is the spring constant of the channel, θ is the twisting angle, L is the channel length, and r is the channel bending radius. The sensor is independent of fluid temperature and pressure, which is one of the device’s main advantages [107].

Q = K_s_θ/(4ωLr) (2)

**Figure 5:**
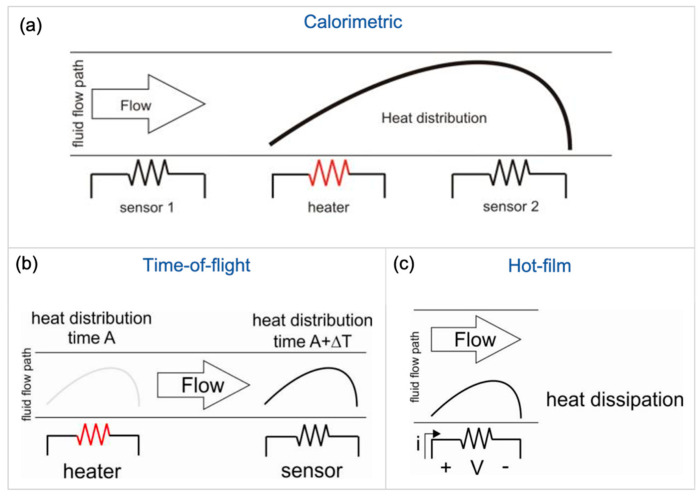
The three types of thermal flow sensors based on their operating mechanisms [108]. (**a**) The Calorimetric flow sensor; (**b**) the time-of-flight thermal flowmeter; and (**c**) is the hot-film sensor, based on heat dissipation.

The thermal flow sensor operates by the heat transfer principle. It consists of a combination of thermosensitive sensors and heaters located on thermally isolated material. The hot-film, calorimetric, and time-of-flight are three common types of thermal flow sensors [108]. The hot-film flowmeter consists of a thin-film resistive heater that dissipates heat to the surrounded fluid. The fluid flow rate is directly proportional to the sensor heat loss that can be detected by the change in the film resistance [109], or voltage change [110], or the required power to keep the heater constant at a specific temperature [111,112]. The calorimetric flow sensor involves a thin film heater located between a minimum of two thermal sensors. The fluid flow rate is determined by studying the heat distribution profile between the thermal sensors before and after the heater [113,114,115,116]. The temperature difference between the upstream and downstream sensors is directly proportional to the flow rate [112]. At flow rate equal to zero, the heat is distributed uniformly to form zero difference between the thermal sensors. The time-of-flight sensor consists of a heater to generate thermal pulses and a temperature sensor located after the heater to detect the generated heat pulses [117]. The time between generating and detecting the heat pulse is inversely proportional to the flow rate. It is also a function of the separated distance between the heater and thermal sensor and the fluid thermal characteristics, such as the thermal conductivity and diffusivity of the fluid. Figure 5 shows the operating principle for the three discussed types of thermal flowmeters [108]. The thermal flow sensors provide high-resolution results with a simple fabrication process. One of the main challenges in the thermal flow sensors is providing an excellent thermal isolation substrate for accurate results and low power consumption. The used silicon wafers as the primary substrate for semiconductor manufacturing processes is a thermally conductive material. That leads to MEMS-thermal flowmeters development as a solution for better thermal isolation, such as locating the thermal sensor on a membrane to improve the thermal isolation [118,119]. Moreover, all the mentioned thermal flow sensors contain at least a heater as an essential element in their design, leading to the high power consumption of the device, in tens mW, which can affect the fluid’s initial temperature [111,120].

A pressure-based flowmeter was developed for the first time for microfluidics in the middle of 1990s [121]. It involves micro-pressure sensors array distributed along the microfluidic channel, such as MEMS, capacitive, and piezoresistive pressure sensors. Then, the flowrate is detected from the absolute pressure change or by studying the pressure distribution along the micro-channel, or by measuring the pressure difference between two pressure sensors [55,122,123]. Pressure-based flowmeters for microfluidic channels have low power consumption compared to other technologies, where it consumes power less than 30 μW [119].

Optical techniques have been used in a variety of flowmeters applications. It employed in MEMS sensors for detecting the deflected micromachined structures, such as a cantilever or a membrane [122,124]. Other optical flowmeters are based on studying the shined light into, out of, and reflected from a transparent planer microfluidic channel. Moreover, optical microfibers have been developed in the last decade in microfluidics flowmeters, by integrating the microfiber into microchannel for light-fluid coupling and interactions [125]. The hot wire or the heat transfer effect using the microfiber optics to generate heat into the microfluidic channel, where the heat loss is directly proportional to the fluid flow rates that can be translated into a spectrum shift [126,127]. Additionally, fiber optics have been utilized in a confined microparticle flowmeter. The microparticle is trapped between the flow force and the optical force, where the manipulation length between the microparticle and the fiber optics edge is inversely proportional to the channel flow rate [128,129]. Optical sensors provide high resolutions even in low flow rates, such as nL/min. However, optical instruments are sophisticated and consist of expensive instruments [130]. 

MEMS, thermal, and pressure-based flowmeters are some examples of the microchannel fluidic flowmeter technologies might be the solution for macro tubular applications in the hopes of overcoming the main challenges of the existing flow sensors. The micro-channel flowmeter technologies provide several advantages over conventional sensors, such as progressing reliability, performance, functionality, and lowering the cost by decreasing the device dimensions to microscale [85]. Table 2 shows a comparison of different flowmeters technologies for microfluidic applications.

## 4. Viscosity Sensors 

Viscosity is the ratio of the shearing stress to the velocity gradient in a fluid. It is a physicochemical measurement for fluid analysis that is a critical indicator for most industries. It plays an important role wherever fluid exists, such as in food industries [87,88], the oil and gas [89], chemical [91,92], and medical industries [131]. Viscosity measurements are a quality control factor for fluids [132], where it used to ensure the fluid consistency of products. Moreover, in the fluid transportation sectors of industries, measuring the fluid viscosity helps in designing the production and transportation processes, such as the required types of pumps, pumping power, energy consumption, and the wall thickness of a pipe system [133,134]. Furthermore, it is essential for the machinery maintenance sector [135]. The viscosity measurement of lubricant oils provides degradation information about machinery oils to determine the health condition of the oil and machine [136]. Specifically, when the lubricant oil is no longer achieving its function and needs to be replaced. Therefore, viscosity sensors help engines to run at optimal efficiency, increase their lifetime, and reduce maintenance costs by affording early alarms, avoiding unnecessary frequent oil replacement, or minimizing machine shutdowns [137]. Hence, the real-time viscosity sensors provide support for low maintenance costs, high-quality control, and efficient management.

Viscosity is usually measured using an off-line laboratory setup [138], such as capillary viscometer [139] and mechanical based measuring principles. One of the most reliable methods for viscosity measurements is the laboratory capillary viscometer, and it is the standard methodology used, due to its extreme accuracy [139]. Capillary viscometers work by measuring the fluid flowrate or the time it takes for a known volume of fluid to flow through a capillary with a fixed length and diameter, or by comparing the measured pressures required to keep a fluid moving with a constant flow rate through capillaries with same diameter and varied lengths [140,141,142]. Some examples of mechanical viscometer techniques are rotational movements [143,144], falling objects viscometer [145,146,147], and vibrational sensors [148,149,150]. Another laboratory setup is the rotational based viscometer that determines the viscosity from the measured torque change of a body with known mass and dimensions rotating at a constant speed in the test fluid [151,152]. The falling object-based technique determines the viscosity by correlating the time it takes for an object, such as a sphere or cylinder, with known mass and volume to fall a fixed distance through the fluid medium. Finally, vibrational viscometers determine the viscosity by measuring the damping of an oscillating resonator within the test fluid. Although the above mentioned off-line laboratory measurements provide accurate results, these methods require a large volume of a fluid sample, are time-consuming, and are designed for a laboratory setup with costly and delicate equipment. Table 3 presents the off-line laboratory viscometers comparison.

Based on traditional off-line laboratory measurements, there are several measuring approaches for in-line or on-site viscometers in tubular systems, such as rotational, vibrational [153], and tube velocity profile methods [154,155,156]. The rotational in-line viscometer operates by measuring the change in torque for a rotational body, as explained in the previous paragraph, only now directly immersed in fluid stream. The vibrational in-line sensor determines the viscosity by monitoring the variation in the damping factor for the resonating probe immersed in the fluid. The main advantage of the vibrational method is the lack of moving parts and operates even under high pressure [156]. However, these methods are bulky and rigid that might disturb the fluid flow and generate a significant pressure drop. In addition, they are destructive and invasive methods, which means they cause permeant damage to the tubular systems during the installation process. Attempts have been made to overcome these challenges by using a combination of flow and pressure sensors, known as the tube velocity profile method. This measures viscosity of laminar flows in known dimensions of a tube by determining the fluid velocity and the pressure drop at a specified length. It can be a non-invasive method when using non-destructive flow sensors, such as ultrasonic flowmeters, for measuring in-line viscosities [157,158,159]. This technique is inexpensive and easy to implement, but the main drawback is that it is restricted to laminar flows only [156]. Although it is evident that viscosity testing plays an essential purpose in industries, there are limited methods available for in-line measurements [158]. Table 4 is a review of the in-line monitoring viscometers.

There is still a demand for developing reliable in-line viscometers [160], however, real-time viscometers have been established and developed robustly for microchannels. Therefore, using micro sensing technology for fluid monitoring applications in the macro tubular system might be the solution for a real-time viscometer. The following sections will discuss each of these microchannel technologies, and their potential for use in macro tubular systems. 

### Microfluidic Viscometers 

Real-time viscometers have been established and developed robustly for microchannels. Nowadays, with the development of microfluidics technology, remarkable works have been implemented to support a variety of microchannels applications, such as in chemical, biological [161,162], medical [163], and micro-reactors [164]. The viscometer sensing technologies for microchannels are based on MEMS [165,166] and micro-resonators [167,168], micro-capillary viscometer [161,169,170], optical sensors [171,172], acoustics [173,174], and ultrasonic based sensors [175].

Microchannels frequency-based viscometers are commonly used for on-site measurements in macro tubular applications or in-line for real-time measurements in microfluidic applications, including the surface acoustic wave sensor (SAW), thickness-shear mode (TSM) resonators, and MEMS. The surface acoustic wave sensor (SAW) device consists of two interdigitated electrodes (IDTs), located on a piezoelectric material, separated by an active area for fluid testing [176]. One IDT is an acoustic wave transmitter, and the other IDT is a receiver. The fluid absorbs the acoustic signal based on the dissipation of the fluid viscous energy and thermal conductivity. In other words, the sound mechanical energy is converted into frictional losses during wave motion in the fluid sample. Shear horizontal surface acoustic waves (SH-SAW) viscosity sensor is similar to the SAW sensor, except for the displacement direction of the wave’s components [177,178]. Both sensors, SAW and SH-SAW, are suitable for biomedical applications, with samples having a small volume of fluid and a low viscosity range. They are not useful for high viscous liquid or large fluid volume, due to acoustic signal absorptions before the transmitted wave can reach the IDT receiver of the device. TSM viscometer is notable for its simplicity without moving parts, since it consists of piezoelectric crystal disk, such as AT-cut quartz, with electrodes on both faces. When applying an alternative voltage to the electrodes, the crystal disk vibrates at MHz frequency range [179]. The resonator characteristics for the crystal vary depending on the fluid viscosity range, such as impedance change and frequency shift [179,180,181]. The thickness shear mode (TSM) viscometers are successful for real-time measurements since they operate at a high-frequency resonating range, in MHz [181]. However, this is also the major drawback, as the high-frequency operation range requires a complicated readout electronics circuitry [167]. MEMS viscometers for microchannels, such as the resonating cantilever, bridge, or beam, are also not applicable for tubular applications. The damping factor for MEMS structures decreases with the fluid viscosity, making them challenging for viscous fluids [167,182]. Additionally, MEMS are not preferable for flexible electronics, due to their low reliability under mechanical deformation, such as bending and flexing conditions, which might generate sensor deformation or permanent stiction problems. Therefore, frequency-based viscometers used in microchannels are not feasible for upscaling for macro tubular systems. 

The operating principle for the microchannel capillary viscometer is the same as the conventional laboratory capillary [183]. However, a pumping system is required in a microcapillary viscometer to pump fluids into the channel, unlike a laboratory tubular capillary that depends on gravity to withdraw fluids to the sensing tube. The microfluidic capillary determines the fluid viscosity by measuring the fluid velocity or the pressure drop in a microchannel, based on the Hagen–Poiseuille equation that describes the fluid dynamics for Newtonian laminar flow, as expressed in Equation (3) [184], where μ is the dynamic viscosity, ν is the fluid velocity, ΔP is the pressure drop between two points in the channel, L is the separated distance between them, K is channel geometry constant, $d_{h}$ is the hydraulic diameter of the channel. For the rectangular cross-section channel, the d_h_ = 2hw/(h + w), where w is the channel width and h is the channel height and the K = 32 [152,161,185].

(3) $$\mu \;=\frac{\Delta {P\;d}_{h}{}^{2}\;}{K\;\nu \;L\;}$$

A fluid’s velocity in a microchannel is found by measuring the time required for the liquid to pass the channel, with known dimensions, using the help of a video recorder and microscope. An alternative method is to measure the pressure difference required to keep fluid moving at a constant velocity through a channel of known length, which is related to viscosity as defined in Hagen-Poiseuille’s law [186,187]. Such a method provides accurate results for real-time measurements in microchannels applications. Viscosity-rheometer-on-chip (VROC) is a commercial handheld product for on-site testing developed on a microcapillary operational method [188]. It consists of an array of MEMS pressure sensors located bellow a microchannel. The device contains a syringe pump, to allow for the precise control of fluid flow rates into the microchannel for accurate viscosity determinations. It supplies precise results in near real-time readings for withdrawn fluids from the tubular systems using the microchannel technology. Microchannel capillary viscometers require manual fluid withdraw, followed by fluid pumping using an external pump to the microchannel, and different sets of microchannels for diverse viscosity ranges. It uses costly fabrication processes, such as lithography fabrication process on silicon wafers.

Optical viscometers are frequently used for real-time measurements in microchannels. Some examples of commonly used optical viscometers are optical-capillary [189], fluid fluorescent analysis [190,191,192], and the combination optical-mechanical sensor [171]. Optical sensors provide highly accurate results for real-time viscosity measurements, and, for this reason, are used mostly for the research and studying phase. The optical-capillary is based on measuring the fluid velocity using optical methods for determining the viscosity [193]. They are mainly using the support of optics, such as microscopes [152], digital cameras [194], lasers, and photodiodes [195], to determine the required time for the fluid to pass between two points separated by a known distance. For example, a digital camera placed above a transparent microchannel, video recording the fluid flow, and calculating the displacement time between two chosen points [161]. The viscosity fluorescent sensing methodology is based on analyzing the fluorescent signal from a fluid sample, after exposing the sample to an energy source, such as a laser. A fluid fluorescent lifetime change depends on fluid viscosity [196,197]. This method functions successfully on fluorescing fluids only. The optical-mechanical sensor is a combination of MEMS sensors with optical measuring techniques to detect the mechanical change of the MEMS devices, instead of depending on the MEMS resonating damping factor [171,198]. In summary, all the optical viscometers require a clear, transparent, flat surface container or microchannel for accurate readings, to allow for the transmitting and receiving of optical waves. Unfortunately, this is not the case for conventional tubular systems, which are curved and mostly made from opaque materials such as steel or polymer. Table 5 presents a summary of real-time microfluidic viscometers.

There have been significant achievements in microfluidic applications. Some have been developed for mechanically flexible electronics, and others are used for on-site monitoring, as an application for microfluidics for macro tubular systems for near real-time measurements. The development of a real-time viscometer is still challenging, especially for an integrated sensory system on a flexible platform, to provide surface adaptation with different tubular dimensions and curved structures.

## 5. Conclusions

The real-time monitoring of fluid properties in tubular systems is rapidly growing and capturing the interest of industry. This work discussed the basic requirements for real-time monitoring sensors for tubular systems. The available fluid monitoring mechanisms are still not meeting the industrial standards of low fluid disturbance, the avoidance of significant damage to assets, and the provision of reliable real-time measurements. Various achievements have been presented and some show promise, such as the non-invasive and NDTs, the utilization of microchannel sensors, and flexible electronics for real-time fluid monitoring.

## Figures and Tables

**Table 1:** Comparison among different flowmeters for tubular systems.

	Differential Pressure	Turbine	Electromagnetic	Ultrasonic	Vortex	Coriolis
Real-Time	Yes	Yes	Yes	Yes	Yes	Yes
Pressure Drop	High	High	No	No	High	High
Size	Large	Bulky	Large	Large	Bulky	Bulky
Flexible Electronics	Potential	N/A	Reported	Reported	No	N/A
Invasive	Yes	Yes	No	No	Yes	Yes

**Table 2:** Comparison among several microfluidic flowmeters.

	Pressure Difference	Optical	Thermal	MEMS
Real-Time Measurements	Yes	Yes	Yes	Yes
Tubular Application Potential	Yes	No	Yes	Yes
Flexible Electronics Potential	Yes	No	Yes	No
Power Consumption	Low	High	High	High

**Table 3:** Off-line laboratory viscometers comparison.

	Capillary	Falling Objects	Rotational	Vibrational
Sample Size	L-ml	L-ml	L-ml	ml-μL
Accuracy	Very high	High	High	High
Real-Time Potential	Yes	No	Yes	Yes
Flexible Electronics	Yes	No	No	Yes

**Table 4:** Review of in-line monitoring viscometers.

	Rotational	Vibrational	Tube Velocity
Real-Time	Yes	Yes	Yes
Pressure Drop	Yes	Yes	No
Size	Bulky	Large	Small
Invasive Installation	Yes	Yes	No
Accuracy	High	High	Low
Flexible Electronics Potential	No	Yes	Yes
Flow Type	All	All	Laminar

**Table 5:** Summary of real-time microfluidic viscometers.

	MEMS	TSM	SAW	Optical	Capillary
Real-Time	Yes	Yes	Near	Yes	Yes
Potential for Tubular Application	No	Yes	No	No	Yes
Flexible Electronics	No	Yes	Yes	No	Yes
Power Consumption	High	Low	Low	High	Low
Electronics Complexity	Simple	Complex	Complex	Very complex	Very simple
